# Supplementary material for: GINA Implementation Improves Asthma Symptoms Control and Lung Function: A Five-Year Real-World Follow-Up Study
Source: J Pers Med. 2023 May 10;13(5):809. doi: 10.3390/jpm13050809 (PMC10222601; doi:10.3390/jpm13050809)
Supplement: Supplementary file 1 [file jpm-13-00809-s001.zip › jpm-2372717-supplementary.pdf]

SUPPLEMENTARY

GINA implementation Improves Asthma Symptoms Control and Lung Function: A Five-Year Real-World Follow-Up Study

Nguyen Van Tho [1,2], Vu Tran Thien Quan [2,3], Do Van Dung [4], Nguyen Hoang Phu [5], Anh Tuan Dinh-Xuan [6], Le Thi Tuyet Lan [2,*]

1 Department of Tuberculosis and Lung Diseases, Faculty of Medicine, University of Medicine and Pharmacy at Ho Chi Minh City
2 Department of Pulmonary Functional Exploration, University Medical Center, University of Medicine and Pharmacy at Ho Chi Minh City, Vietnam
3 Department of Physiology, Faculty of Medicine, University of Medicine and Pharmacy at Ho Chi Minh City
4 Department of Biostatistics, Faculty of Public Health, University of Medicine and Pharmacy at Ho Chi Minh City
5 Department of Pulmonology, Dong Nai General Hospital, Bien Hoa City, Vietnam
6 AP-HP, Hôpital Cochin, Service de Physiologie-Explorations Fonctionnelles, Paris, France
* Correspondence: tuyetlanyds@gmail.com; Tel.: +84-028 38594470

Table S1. Level of asthma control according to GINA 2006.

Characteristic	Controlled (All of the following)	Partly controlled (Any present in any week)	Uncontrolled
**Daytime symptoms**	Twice or less per week	More than twice per week	3 or more features of partly controlled asthma present in any week
**Limitations of activities**	None	Any	
**Nocturnal symptoms / awakening**	None	Any	
**Need for rescue / "reliever" treatment**	Twice or less per week	More than twice per week	
**Lung function (PEF or FEV1)**	Normal	< 80% predicted or personal best (if known) on any day	

Table S2. Level of asthma control according to GINA 2016.

Symptom control		Level of asthma symptom control		
In the past 4 weeks, has the patient had:		Well-controlled	Partly controlled	Uncontrolled
Daytime asthma symptoms more than twice a week?	Yes❑ No❑	None of these	1-2 of these	3-4 of these
Any night waking due to asthma?	Yes❑ No❑			
Reliever needed for symptoms more than twice a week?	Yes❑ No❑			
Any activity limitation due to asthma?	Yes❑ No❑			

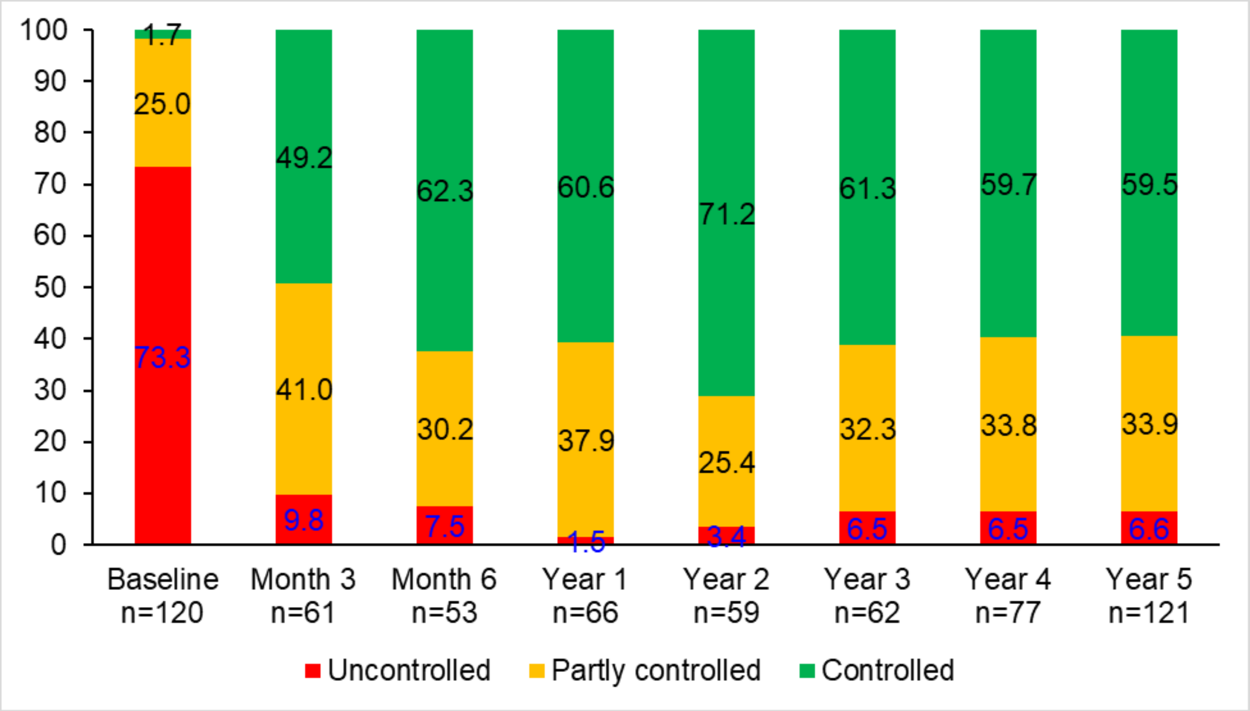

Figure S1. Proportions of different levels of asthma symptoms control during 5 years of asthma management among 121 patients who attended the follow-up visit at year 5.

Table S3. Relation between baseline characteristics and the proportion of well-controlled asthma at year 1.

Baseline characteristics		Well- controlled asthma	P value
Childhood asthma	No (n=536)	343 (64.0%)	0.4515
	Yes (n=232)	155 (66.8%)	
Allergic rhinitis	No (n=549)	360 (65.6%)	0.4563
	Yes (n=220)	138 (62.7%)	
GERD	No (n=670)	437 (65.2%)	0.4854
	Yes (n=99)	61 (61.6%)	
BMI ≥ 25 kg/m2	No (n=670)	438 (65.4%)	0.3574
	Yes (n=99)	60 (60.6%)	
Co-paid by health insurance	No (n=561)	353 (62.9%)	0.1550
	Yes (n=197)	135 (68.5%)	
ICS molecule	Budesonide (n=224)	151 (67.4%)	0.3480
	Fluticasone (n=534)	341 (63.9%)	

Data are presented as n (%). GERD, gastroesophageal reflux; ICS, inhaled corticosteroid.

Table S4. Relation between baseline characteristics and the proportion of well-controlled asthma at year 5.

Baseline characteristics		Well-controlled asthma	P value
Childhood asthma	No (n=82)	49 (59.8%)	0.9348
	Yes (n=39)	23 (59.0%)	
Allergic rhinitis	No (n=78)	46 (59.0%)	0.8729
	Yes (n=43)	26 (60.5%)	
GERD	No (n=101)	63 (62.4%)	0.1517
	Yes (n=20)	9 (45.0%)	
BMI ≥ 25 kg/m2	No (n=104)	65 (62.5%)	0.1002
	Yes (n=17)	7 (41.2%)	
Co-paid by health insurance	No (n=92)	56 (60.9%)	0.4932
	Yes (n=28)	15 (53.6%)	
ICS molecule	Budesonide (n=40)	25 (62.5%)	0.7107
	Fluticasone (n=78)	46 (59.0%)	

Data are presented as n (%). GERD, gastroesophageal reflux; ICS, inhaled corticosteroid.

Table S5. Relation between baseline characteristics and proportion of patients who attended the follow-up visit at year 1.

Baseline characteristics		Follow-up visit at year 1	P value	OR (95% CI)
Gender	Male (n=491)	274 (55.8%)	0.8240	1.03 (0.82-1.28)
	Female (n=897)	495 (55.2%)		
Place of residence	Other provinces (n=932)	537 (57.6%)	**0.0178**	**1.31 (1.05-1.64)**
	Ho Chi Minh City (n=456)	232 (50.9%)		
Childhood asthma	Yes (n=408)	232 (56.9%)	0.4704	1.09 (0.86-1.38)
	No (n=979)	536 (54.8%)		
Allergic rhinitis	Yes (n=357)	220 (61.6%)	**0.0059**	**1.41 (1.10-1.80)**
	No (n=1031)	549 (53.3%)		
GERD	Yes (n=178)	99 (55.6%)	0.9691	1.01 (0.73-1.38)
	No (n=1208)	670 (55.5%)		
BMI ≥ 25 kg/m2	No (n=1182)	670 (56.7%)	**0.0219**	**1.41 (1.05-1.90)**
	Yes (n=206)	99 (48.1%)		
Bonchodilator reversibility	Yes (n=773)	441 (57.1%)	0.1856	1.16 (0.93-1.43)
	No (n=604)	323 (53.5%)		
Co-paid by health insurance	Yes (n=324)	197 (60.8%)	**0.0462**	**1.29 (1.00-1.67)**
	No (n=1029)	561 (54.5%)		
ICS molecule	Fluticasone (n=919)	534 (58.1%)	**0.0111**	**1.34 (1.07-1.69)**
	Budesonide (n=441)	224 (50.8%)		
High-dose ICS	Yes (n=1050)	606 (57.7%)	0.0558	1.30 (0.99-1.69)
	No (n=275)	141 (51.3%)		

GERD, gastroesophageal reflux; ICS, inhaled corticosteroid.

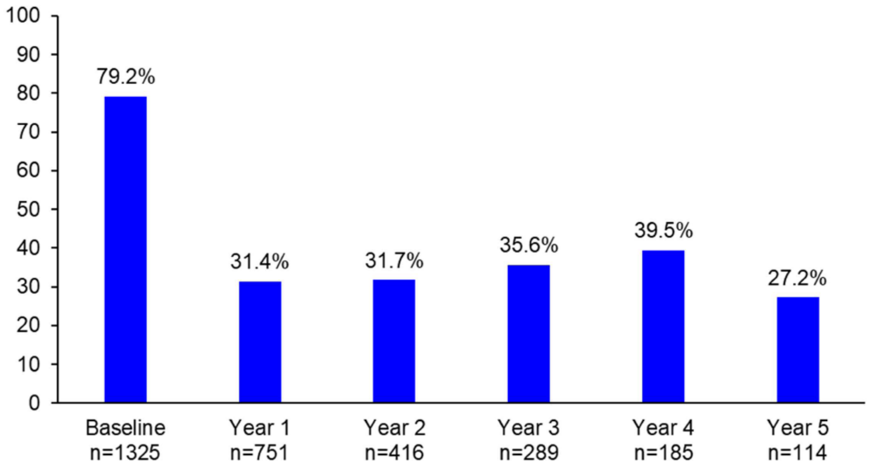

Figure S2. The proportion of patients with high-dose ICS decreased over 5 years of asthma management. High-dose ICS was defined as equivalent fluticasone propionate > 500 μg/day.
